# Evaluation of anti-COVID-19 measures taken by the parents of children with celiac disease: a cross-sectional study

**DOI:** 10.1590/1516-3180.2020.0644.10122020

**Published:** 2021-03-03

**Authors:** Ayşegül Bükülmez, Melike Taşdelen Baş, Esra Çiftci

**Affiliations:** I MD. Physician and Professor, Department of Pediatrics, Afyonkarahisar Health Sciences University, Afyonkarahisar, Turkey.; II PhD. Nurse and Lecturer, Selcuk University Aksehir Kadir Yallagoz Health School, Konya, Turkey.; III MD. Pediatrician, Department of Pediatrics, Afyonkarahisar Health Sciences University, Afyonkarahisar, Turkey.

**Keywords:** COVID-19 [supplementary concept], Celiac disease, Parents, Public health, Infection control, Children, Nutrition, Prevention.

## Abstract

**BACKGROUND:**

Coronavirus disease 2019 (COVID-19) causes negative life changes brought about through lockdowns, in addition to severe complications and death. Among these changes, asplenism or hyposplenism has been reported in patients with celiac disease. It has been reported that the risk of pneumococcal sepsis is higher in celiac patients with hyposplenism. Moreover, celiac patients present high risk of admission to hospital due to influenza.

**OBJECTIVE:**

To determine the degree of awareness of COVID-19 among parents of children with celiac disease and examine the measures that they take.

**DESIGN AND SETTING:**

Cross-sectional study at a university hospital in the Middle Anatolian region of Turkey.

**METHODS:**

The diagnosis of celiac disease was confirmed through a survey conducted online among 73 parents between May and July 2020.

**RESULTS:**

The mean age was 37.57 ± 6.56 years for the mothers, 41.15 ± 5.56 years for the fathers and 11.36 ± 4.36 years for the children. 90.4% of the parents reported that COVID-19 was transmitted through “speaking, coughing, sneezing and infection of the face after contact with virus-infected surfaces”. Moreover, 78.1% indicated that they did not have any difficulty in finding gluten-free foods.

**CONCLUSION:**

These parents of children with celiac disease believed that their children’s risk of developing COVID-19 did not differ from that of healthy children. It was also observed that appetite and states of nervousness were higher among these children with celiac disease during lockdowns and that their sleep patterns were affected.

## INTRODUCTION

Coronavirus disease (COVID-19) first emerged in the city of Wuhan, China, in December 2019 and has spread all over the world. Its causative factor is a ribonucleic acid (RNA) virus called severe acute respiratory syndrome coronavirus-2 (SARS-CoV-2) which is similar to SARS-CoV.^1^^,^^2^

Although the mortality rate for COVID-19 ranges between 1.4 and 7.2%, the rates have been found to be higher among individuals who have underlying comorbid diseases.^1^^,^^2^While COVID-19 has primarily been characterized by the respiratory impact of viral pneumonia, it affects multiple organ systems with significant morbidity and mortality among critically ill patients.^3^

Coronavirus disease 2019 (COVID-19) is seen in all age groups. Its clinical course and prognosis are relatively mild in children, compared with adults. It may result in mortality among adults, especially those who have underlying comorbid disease such as diabetes and cancer.^4^^,^^5^ All countries have taken serious measures in line with the recommendations of the World Health Organization (WHO), including lockdowns,social distancing, hand hygiene, mask use and limitations of hospital visits in order to prevent the spread of this disease. There is no clear evidence regarding how lockdowns affect quality of life, especially among children and individuals with chronic diseases.^1^^,^^6^

The World Health Organization has reported that the coronavirus causing COVID-19 is transmitted through droplets and by touching one’s mouth, nose or conjunctiva after touching virus-contaminated surfaces. Moreover, it can be transmitted on objects that have been used by an infected person.^1^^,^^7^ For working individuals, it is important to regularly clean the working environment and the keyboards, tables and telephones that are used in the workplace.^1^

Celiac disease is an autoimmune enteropathy caused by the immune reaction that is developed through gluten intake among individuals who have a genetic predisposition to this. The treatment for celiac disease consists of a lifelong gluten-free diet.^8^^,^^9^ Since celiac patients may have difficulty in finding gluten-free foods during lockdownperiods, asking for support from self-care agencies is recommended.^10^

The Celiac Disease Foundation has proposed that, in the present pandemic situation, individuals with celiac disease should maintain stocks of gluten-free foods and should make gluten-free meal plans and train themselves to use new gluten-free recipes. Moreover, this foundation has underlined the need for celiac patients to stay away from crowds and to stay at home, in order to minimize their exposure to the virus.^11^ Defective spleen function is frequently seen in autoimmune gastrointestinal diseases such as celiac disease. As it is well known that celiac patients are at high risk of developing sepsis with encapsulated bacteria, pneumococcal vaccination is recommended.^9^^,^^12^Asplenism or hyposplenism has been also reported in celiac patients, along with gastrointestinal symptoms. It has also been reported that the risk of pneumococcal sepsis is higher in celiac patients with hyposplenism.^13^Mårild et al. reported that celiac patients were at higher risk of hospital admissions due to influenza. Celiac patients experience anxiety and depression due to the increased risk of infection.^14^ Moreover, lockdown conditions and limitations during the pandemic, along with fear of the difficulties that may be experienced in trying to find gluten-free foods, increase anxiety.

Celiac disease is a chronic disease that has leads to the risk of sepsis and hospitalizations due to influenza, and the risk of pneumococcal sepsis due to hyposplenism that patients may experience. For this reason, patients with celiac disease need to be more careful in terms of the precautions to be taken in preventing COVID-19, which is known to cause severe complications. Since the daily life requirements of children with celiac disease will be controlled by their parents, the measures taken by parents for their children were evaluated in this study.

## OBJECTIVE

This study was carried out to determine the degree of awareness of COVID-19 among the parents of children with celiac disease and the measures that they take.

## METHODS

This was a cross-sectional study that was conducted among the parents of patients aged 4-18 years who had been diagnosed with celiac disease in the pediatric gastroenterology outpatient clinic of a health sciences university in Afyonkarahisar, Turkey. The diagnosis of celiac disease was made based on serological tests to detect immunoglobulin A (IgA)-tissue transglutaminase IgA (tTGA) and/or immunoglobulin G (IgG)-tissue transglutaminase IgG (tTGG); and on and histological examination of small-intestine mucosal biopsies. Data for this study were collected by means of a questionnaire, which was generated online and could be completed within 10 minutes, between May 2020 and July 2020.

We arbitrarily decided to include the parents of patients with celiac disease who were undergoing regular follow-up at the study hospital. We included the mothers or fathers of children in the 0-18 age group with celiac disease. We collected data using a semi-structured questionnaire (this questionnaire is presented in the Appendix). The questionnaire was created based on up-to-date information from the literature. The parents who agreed to participate in the study were asked to fill out a demographic information form consisting of 17 questions and an information form relating to COVID-19 infection that included 34 questions. Although our aim was to reach a total of 150 parents, only 73 parents who agreed to participate and who were contacted through the internet were included in the study.

The data obtained were assessed by means of descriptive statistics (arithmetic mean, median, standard deviation and percentage distribution). Means were compared between groups and the compliance of the data with normal distribution was analyzed using the Shapiro-Wilk test. An independent-sample t test was used for parametric data and the Mann-Whitney U test was used for nonparametric data. The chi-square test was used to compare percentage distributions of categorical variables between groups. The analyses on the data were conducted using the IBM SPSS Statistics™ software, version 20.0 (IBM Corporation, Armonk, NY). The results were analyzed within a 95% confidence interval and P < 0.05 was considered to be statistically significant.

Approval for this study was obtained from the clinical research ethics committee of the local medical school (5.5.20/ 2011-KAEK-2/2020/186). All the participants in this study gave written consent for their inclusion.

## RESULTS

Among the parents included in the study, 42 (57.5%) were mothers and 31 (42.4%) were fathers. Out of the children with celiac disease, 45 (61.6%) were females and 28 (38.4%) were males and their mean age was 11.36 ± 4.36 years. In terms of age group, 22 (30.1%) were 4-8 years, 21 (28.8%) were 9-12 years and 30 (41.1%) were 13-18 years. The children’s ages at diagnosis were found to be 1-4 years in 48 cases (65.8%), 5-8 years in 23 cases (31.5%) and 9-11 years in two cases (2.7%). The patients’ mean height was 139.97 centimeters (range, 100-168 centimeters) and their mean weight was 35.7 kilograms (range, 13-70 kilograms). Within the last month, 15 patients (20.5%) had had a weight gain of less than one kilogram, 17 patients (23.3%) had not had any weight change, 39 patients (53.4%) had had a weight gain of more than one kilogram and two patients (2.7%) had had a weight loss of more than one kilogram.

Among the families, 24 (32.9%) were living in a village, 14 (19.2%) were living in a town and 35 (47.9%) were living in a city center. Sixty-seven families (91.8%) were nuclear families and 6 (8.2%) were extended families. Among all the families participating in this study, four (5.5%) had one child, 28 (38.4%) had two children, 30 (41.1%) had three children and 11 (15.1%) had four or more children. Fifty-two families (71.2%) had health insurance and 21 (28.8%) did not. Thirty-four families (46.6%) indicated that they had experienced a loss of income during this pandemic period. The parents’ education levels and employment statuses are shown in Table 1.

**Table 1.** Sociodemographic characteristics of the parents

Education level	Mother (n = 42)	Father (n = 31)
Literate/elementary school	22 (52.3%)	5 (16.1%)
Secondary school/high school	14 (33.3%)	21 (67.7%)
University/higher	6 (14.2%)	5 (16.1%)
Employment status of parents		
Office employee	9 (21.4%)	10 (32.2%)
Other employees	14 (33.3%)	11 (35.4%)
Self-employed	10 (23.8%)	1 (3.2%)
Retired	1 (2.3%)	3 (9.6%)
Unemployed	7 (16.6%)	6 (19.2%)

In response to the question “Do you think that your child has a greater risk of developing coronavirus disease (COVID-19) compared with other children?”, 53.4% of the parents answered “yes” and 46.6% answered “no”. No statistically significant difference was found between the mothers and fathers (P = 0.46).

The parents’ answers to the question “How is COVID-19 transmitted?” were “coughing” (2; 2.7%), “transmission after contact with virus-contaminated surfaces” (5; 6.8%) and “speaking, coughing, sneezing and contamination of the face after contact with virus-contaminated surfaces” (66; 90.4%).

“How should your child behave while coughing or sneezing?” was answered as “use the area inside the elbow while sneezing” by 75.3%, “use a napkin or handkerchief if available” by 23.3% and “use the back of the hand” by 1.4% of the parents.

19.2% of the individuals participating in the survey were found to have pets in their homes. They answered the question “Is coronavirus disease (COVID-19) transmitted from pets?” as “yes” by 37% and “no” by 63%.

The question “Which of the following precautions is effective in preventing transmission of coronavirus disease (COVID-19)?” was responded as “washing hands frequently with soap and water” by 17.8%, “staying away from people showing flu-like symptoms” by 1.4% and “washing hands frequently with soap and water, cleaning hands with alcohol-based disinfectants, avoiding face-to-face contact and staying away from people showing flu-like symptoms” by 80.8%.

The question “Which age groups are affected by coronavirus disease (COVID-19)?” was answered as “over 65 years old” by 23.3%, “20-65 years old” by 6.8% and “all age groups” by 69.9%. “Is the spread of coronavirus disease (COVID-19) affected by air temperature change?” was answered as “yes” by 93.4% and as “no” by 6.6% of the participants.

“What is the minimum alcohol content (%) that disinfectants need to have in order to kill the viral factor that causes coronavirus disease (COVID-19)?” was answered as “disinfectants containing at least 70% alcohol” by 72.6%.

“Is there a specific medication to treat coronavirus disease (COVID-19)?” was answered as “no” by 89% of the participants.

The question “For at least how long should hands be washed in order to kill the viral factor that causes coronavirus disease (COVID-19)?” was answered as “20 seconds” by 94.5%.

“What do you use to clean common areas in your home (tables, stairs, door handles, light switches, counters, toilets, taps and sinks) in order to prevent transmission of coronavirus disease (COVID-19)?” was answered as “bleach” by 57.5%, “disinfectants containing 70% alcohol” by 19.2% and “vinegar” by 23.3%.

“What have you been using to clean electronic devices in your home during the coronavirus disease (COVID-19) pandemic?” was answered as “1/100 diluted bleach” by 24.7%, disinfectants containing 70% alcohol” by 23.3%, “spray containing alcohol” by 13.7% and “vinegar” by 38.4%.

The question “What have you been using to clean places such as the toilet and bathroom in your home during the coronavirus disease (COVID-19) pandemic?” was answered as “1/100 diluted bleach” by 97.3% and “disinfectant containing 70% alcohol” by 2.7%.

The question “Have you been going out from your home for any reasons other than your urgent needs or for work during the coronavirus disease (COVID-19) pandemic?” was answered as “no” by 50.7% of the participants.

“Have you been allowing your child to go out from your home during the coronavirus disease (COVID-19) pandemic?” was answered as “no” by 78.1% of the parents included in the study.

“From where do you mostly get news about the coronavirus disease (COVID-19) pandemic?” was answered as “television” by 68.5, “internet” by 30.1% and “I do not follow the news” by 1.1%.

The question “Do you clean materials bought or brought from outside before using them, because of the coronavirus disease (COVID-19) pandemic?” was answered as “yes” by 95.9%.

“Have you been paying attention to social distancing rules during interactions with individuals outside your home during the coronavirus disease (COVID-19) pandemic?” was answered as “yes” by 98.6%.

“Have you been using a mask while going out during the coronavirus disease (COVID-19) pandemic?” was answered as “yes” by 98.6% of the parents. No relationship could be found between the education levels of the parents and their responses (P > 0.46).

“What should be the social distance in order to prevent transmission of coronavirus disease (COVID-19)?” was answered as “1-2 meters” by 97.3%.

“Have you been having any problems in finding foods for your child (gluten-free foods) during the coronavirus disease (COVID-19) pandemic?” was answered as “no” by 78.1% of the parents and “yes” by 29.9%.

“Do you believe that it is important to pay attention to your children’s diet in order to strengthen their immune systems and protect them from coronavirus disease (COVID-19)?” was answered as “yes” by all of the parents.

In relation to the question “Have you been giving vitamin-mineral supplementation to your children during the coronavirus disease (COVID-19) pandemic?”, 26 individuals (35.6%) said that they were not giving any vitamin-mineral supplementation to their children. Administration of propolis was reported by 16 participants (21%), vitamin C by 20 (27.3%), fish oil by 2 (2.7%) and iron by two (2.7%). Moreover, five (6.8%) reported giving their children both propolis and fish oil, one (1.3%) reported giving both carob extract and vitamin C and one (1.3%) reported giving propolis, vitamin C and vitamin D.

In the question “Which foods do you consider to be good for the immune system?”, the responses were the following: garlic (stated by 79.4% of the parents), kefir (a fermented milk drink) (60.2%), oranges/kiwi fruit (57.5%), spinach (56.16%), oily fish (52%), broccoli (49.3%), pickles (47.9%), ginger (45.2%), turmeric(34.2%), red peppers(26%), green tea (17.8%), blueberries(15.1%), potatoes(10.9%) and bitter chocolate (6.8%).

“Which of the following symptoms increased in your child with the closure of schools due to coronavirus disease (COVID-19)?” was answered as “no complaint” by 42 (57.5%), “state of nervousness” by 24.6%, “increase in appetite” by 13.6%, “lack of appetite” by 8.21%, “headache” by 6.84%, “stomach ache” by 4.1%, “increase in hair loss” by 2.73%, “allergic skin rash” by 2.73%, “having nightmares and scary dreams” by 1.36%, “nausea” by 1.36% and “constipation” by 1.36%. There were no mentions of abdominal distension, diarrhea or vomiting complaints in the answers.

“Has your child had any sickness requiring medication (like antibiotics), such as flu, cold or urinary tract infection, in the past month?” was answered as “no” by 94.5%.

“Has your child’s sleep pattern and quality changed in the past month?” was answered as “increase in sleep duration” by 8.2% of the parents, “decrease in sleep duration” by 6.8%, “change in sleep pattern” by 43.8% and “no effect on sleep pattern” by 41.1%.

Among the answers given by the parents to the questions regarding COVID-19, a statistically significant difference was found only in relation to their preference for mask use, and this is shown in Table 2. The parents stated that they had not had their children vaccinated against the flu or pneumococcus in the last year.

The details of the survey questionnaire and survey responses are shown in the Appendix.

**Table 2.** Comparison of the answers given by the parents to the questions regarding COVID-19 disease

	Mother		Father		P^*^
	Yes	No	Yes	No	
Do you think that your child is at greater risk of developing coronavirus disease (COVID-19), compared with other children?^1^	24 57.1%	18 42.9%	15 48.4%	16 51.6%	0.459
Have you been allowing your child to go out during the coronavirus disease (COVID-19) pandemic?^18^	8 19.0%	34 81.0%	8 25.8%	23 74.2%	0.490
Have you been making your child wear a mask when you have needed to take your child out during the coronavirus disease (COVID-19) pandemic?^25^	35 83.3%	7 16.7%	31 100%	0 0.0%	0.017

^*^Chi-square test.

## DISCUSSION

Coronavirus disease 2019 (COVID-19) is a severe acute respiratory syndrome caused by severe acute respiratory syndrome coronavirus-2 (SARS-CoV-2), and it has become a global health crisis through spreading all over the world very quickly. Pediatric patients have constituted 1-5% of the patients diagnosed with COVID-19. All countries have taken serious measures, most notably consisting of lockdowns, in order to prevent the spread of this disease.^15^^,^^16^

This study is the first in the literature to evaluate the measures taken by the parents of children with celiac disease with regard to COVID-19 infection. 90.4% of the parents stated that COVID-19 disease was transmitted through “speaking, coughing, sneezing and face contamination after contact with virus-contaminated surfaces”. Pal et al. reported that 83% of the young adults with type 1 diabetes mellitus who they surveyed thought that COVID-19 disease was transmitted through droplets.^17^

Fifty-nine parents (80.8%) indicated that “washing hands frequently with soap and water, cleaning the hands with alcohol-based disinfectants, avoiding face contact and staying away from people with flu-like symptoms” were important for prevention of transmission of COVID-19. Moreover, washing hands “for 20 seconds” was reported to be important by 94.5% of them. In the study by Pal et al., 53% of the participants stated that staying at home, ensuring that they paid attention to social distancing and doing regular hand washing were important for prevention of transmission of COVID-19.^17^72.6% of the parents in the present study stated that “disinfectants containing at least 70% alcohol” should be used in order to kill coronavirus, and 57.5% indicated that they cleaned common areas at home (tables, chairs, door handles, light switches, counters, toilets, taps and sinks) with bleach in order to prevent transmission of the pandemic. These parents of children with celiac disease also reported that their children’s anxiety about coronavirus contamination was not so different from the concerns of healthy children.

They also reported that they had not had their children vaccinated against flu or pneumococcus over the last year. Siniscalchi et al. reported in their study conducted in Italy that only one child with celiac disease got vaccinated against flu.^18^

In our study, we found that 78.1% of the parents of celiac patients did not experience any problems in finding gluten-free foods for their children during the lockdown, and they did not have any difficulties in complying with their children’s gluten-free dietary needs. Since routine daily activities such as going to school were interrupted during the lockdown, the children became bored. With boredom, they found solace through foods rich in fat, carbohydrates and proteins, which supplied them with large amounts of calories. Furthermore, hearing or reading news about the pandemic created distress and increased their likelihood of consuming certain foods.^19^ Excessive intake of macronutrients during a lockdown situation causes lack of micronutrients and development of obesity.

We also found that 53.4% of the children with celiac disease had had a weight gain of more than one kilogram in the last month. Pietrobelli et al. reported that there was a tendency towards weight gain among 41 children who had stayed at home and had experienced lifestyle changes for three weeks due to a lockdown during COVID-19 pandemic.^20^

Most of the parents indicated that they continued to give propolis and vitamin C supplementation to their children during this period. Furthermore, carob extract, vitamin D, fish oil and iron were among the supplements preferred by the families. Most of the parents reported that they were giving food supplements to their children in accordance with their doctors’ advice.

Supplementation of individuals’ general nutritional status through vitamins and minerals may affect the functioning of their immune system either positively or negatively. Supplementing diet with nutrients such as vitamin D and zinc may alter immune functions.^21^ It has become known that antioxidants increase T cell subgroups, empower lymphocyte responses against mitogens and enhance interleukin 2 (IL-2) production, the number of natural killer cells and the response to influenza vaccines.

Vitamin D decreases the possibility of occurrence of a cytokine storm through protecting tight junctions in the respiratory system, increases virus death through induction of cathelicidin and defensin and decreases the production of proinflammatory cytokines. Thus, it is recommended that patients with celiac disease should take vitamin D supplementation and should consume foods containing vitamin D. The parents surveyed in the present study indicated that they preferred mainly garlic, kefir, oranges, kiwi fruit, spinach, fish, broccoli and pickles for strengthening their children’s immune systems.

Taking immune-boosting foods and balanced planning of meals and portions have been also recommended for patients with celiac disease. Especially cellular immunity, phagocyte function, cytokine production, antibody response, antibody affinity and disturbance in complement system make patients with celiac disease more susceptible to viral infections. During a pandemic situation, consumption of foods containing high amounts of antioxidants, vitamins and minerals strengthens the immune system.^19^

In this study, 58.9% of the parents surveyed here stated that their children’s sleep patterns changed. It has been reported that social isolation during lockdowns causes sleep disorders through increasing anxiety and stress and that this also increases food intake.^19^^,^^22^

In this study, we evaluated the parents’ levels of knowledge about COVID-19 and the measures that they took in relation to their children. In addition, we evaluated these children’s access to a gluten-free diet within their daily lives and their sleep and nutrition patterns during this period. The questions were multiple-choice and open-ended, so as not to affect the answers given by the parents.

The limitations of this study were the small sample size, the lack of a control group and the fact that it was conducted in a single center. We were aware that parents’ feelings about their children’s behaviors would possibly be heightened and that, therefore, their answers might be unintentionally biased. Therefore, we avoided asking directional questions.

## CONCLUSION

This study is important with regard to determining the awareness of COVID-19 among parents who have children with celiac disease and examining the precautions that they take. The results from this study showed that these parents thought that their children with celiac disease did not present a risk of getting COVID-19 that differed from the risk among healthy children. This finding revealed that the parents of children with celiac disease should be informed more about the COVID-19 pandemic.

It was seen that the children’s state of nervousness and associated state of weight gain were found to be affected by the lockdown that was applied to prevent the spread of COVID-19. This lockdown situation had a negative effect on the conditions required to maintain a healthy lifestyle.

## Appendix. Survey Questionnaire and Survey Responses

**Table:**

1. Do you think that your child is at greater risk of developing coronavirus disease (COVID-19), compared with other children?		
Yes	39	53.4%
No	34	46.6%
2. How is COVID-19 disease transmitted?		
Coughing	2	2.7%
Transmission after contact with virus-contaminated surfaces	5	6.8%
Speaking, coughing, sneezing and contamination of the face after contact with virus-contaminated surfaces	66	90.4%
3. How should your child behave while coughing or sneezing?		
Use the area inside the elbow while sneezing	55	75.3%
Use a napkin or handkerchief if available	17	23.3%
Use the back of the hand	1	1.4%
Use palm	0	0%
4. Do you keep pets at home?		
Yes	14	19.2%
No	59	80.8%
5. Is coronavirus disease (COVID-19) transmitted from pets?		
Yes	27	37%
No	46	63%
6. Which of the following precautions is effective in preventing transmission of coronavirus disease (COVID-19)?		
Washing hands frequently with soap and water	13	17.8%
Cleaning hands with alcohol-based disinfectants	0	0%
Avoiding contact with face	0	0%
Staying away from people showing flu-like symptoms	1	1.4%
Washing hands frequently with soap and water, cleaning hands with alcohol-based disinfectants, avoiding face-to-face contact and staying away from people showing flu-like symptoms	59	80.8%
7. Which age groups are affected by coronavirus disease (COVID-19)?		
Over 65 years old	17	23.3%
20-65 years old	5	6.8%
Under 20 years old	0	0%
All age groups	51	69.9%
8. Is any member of your family over 65 years old?		
Yes	68	93.2%
No	5	6.8%
9. Is the spread of coronavirus disease (COVID-19) affectedby air temperature change?		
Yes	34	46.6%
No	39	53.4%
10. What is the minimum alcohol content (%) that disinfectants need to have in order to kill the viral factor that causes coronavirus disease (COVID-19)?		
50%	14	19.2%
60%	6	8.2%
70%	53	72.6%
11. Is there a specific medication to treat coronavirus disease (COVID-19)?		
Yes	8	11%
No	65	89%
12. For at least how long should hands be washed in order to kill the viral factor that causes coronavirus disease (COVID-19)?		
5 seconds	1	1.4%
10 seconds	1	1.4%
15 seconds	2	2.7%
20 seconds	69	94.5%
13. What do you use to clean common areas in your home (tables, stairs, doorhandles, light switches, counters, toilets, taps and sinks) in order to prevent transmission of coronavirus disease (COVID-19)?		
Bleach	42	57.5%
Disinfectants containing 70% alcohol	14	19.2%
Vinegar	17	23.3%
14. What have you been using to clean electronic devices in your home during the coronavirus disease (COVID-19) pandemic?		
1/100 diluted bleach	18	24.7%
Disinfectants containing 70% alcohol	17	23.3%
Spray containing alcohol	10	13.7%
Vinegar	28	38.4%
15. What have you been using to clean places such as the toilet and bathroom in your home during the coronavirus disease (COVID-19) pandemic?		
1/100 diluted bleach	71	97.3%
Disinfectants containing 70% alcohol	2	2.7%
Alcohol containing spray	0	0%
Vinegar	0	0%
16. Should the bleach used for surfaces and toilets be diluted at the same rate as used for disinfection, when used for prevention of transmission of coronavirus disease (COVID-19) disease?		
Yes	31	42.5%
No	42	57.5%
17. Have you been going out from your home for any reasons other than your urgent needs or for work during the coronavirus disease (COVID-19) pandemic?		
Yes	36	49.3%
No	37	50.7%
18. Have you been allowing your child to go out from your home during the coronavirus disease (COVID-19) pandemic?		
Yes	16	21.9%
No	57	78.1%
19. From where do you mostly get news about the coronavirus disease (COVID-19) pandemic?		
Television	50	68.5%
Internet	22	30.1%
Friends	0	0%
Newspaper	0	0%
I do not follow the news	1	1.4%
20. Do you clean materials bought or brought from outside before using them, because of the coronavirus disease (COVID-19) pandemic?		
Yes	70	95.9%
No	3	4.1%
21. How do you clean the materials purchased from outside? (If you answered yes to question 20, please answer this)		
By wiping the packaging boxes with diluted bleach	20	27.4%
By only bringing them inside after they have been kept outside for a day	25	34.2%
By changing the bags from outside	20	27.4%
By wiping with soapy water	2	2.7%
By wiping the surface with disinfectant	3	4.1%
By washing with plenty of water	2	2.7%
By cleansing with vinegar	1	1.4%
22. Have you been paying attention to social distancing rules during interactions with individuals outside your home during the coronavirus disease (COVID-19) pandemic?		
Yes	72	98.6%
No	1	1.4%
23. Have you been using a mask while going out during the coronavirus disease (COVID-19) pandemic?		
Yes	72	98.6%
No	1	1.4%
24. What should be the social distance in order to prevent transmission of coronavirus disease (COVID-19)?		
1-2 meters	71	97.3%
50 cm	2	2.7%
25. Have you been making your child wear a mask when you have needed to take your child out during the coronavirus disease (COVID-19) pandemic?		
Yes	66	90.4%
No	7	9.6%
26. Have you been having any problems in finding foods for your child (gluten-free foods) during the coronavirus disease (COVID-19) pandemic?		
Yes	41	56.2%
No	32	43.8%
27. Do you believe that it is important to pay attention to your children’s diet in order to strengthen their immune systems and to protect them from coronavirus disease (COVID-19)?		
Yes	73	100%
No	0	0%
28. Have you been giving vitamin-mineral supplementation to your children during the coronavirus disease (COVID-19) pandemic?		
Yes	46	63%
No	26	35.6%
29. If your answer to question 28 was yes, what have you been giving and how much?		
Propolis	16	21%
Vitamin C	20	27.3%
Fish oil	2	2.7%
Iron	2	2.7%
Fish oil and propolis	5	6,8%
Carob extract and vitamin C	1	1.3%
Propolis and vitamin C and vitamin D	1	1.3%
30. Which of the following foods do you think are good for the immune system?		
Blueberries	11/73	15.1%
Bitter chocolate	5/73	6.8%
Turmeric	25/73	34.2%
Oily fish	38/73	52%
Broccoli	36/73	49.3%
Spinach	41/73	56.16%
Green tea	13/73	17.8%
Garlic	58/73	79.4%
Kefir (a fermented milk drink)	44/73	60.2%
Oranges or kiwi fruit	42/73	57.5%
Ginger	33/73	45.2%
Red peppers	19/73	26%
Pickles	35/73	47.9%
Potatoes	8/73	10.9%
31. Which of the following symptoms increased in your child with the closure of schools due to coronavirus disease (COVID-19)?		
No complaint	42/73	57.5%
State of nervousness	18/73	24.6%
Increase in appetite	10/73	13.6%
Lack of appetite	6/73	8,21%
Headache	5/73	6.84%
Stomach ache	3/73	4.1%
Increase in hair loss	2/73	2.73%
Allergic skin rash	2/73	2.73%
Having nightmares and scary dreams	1/73	1.36%
Nausea	1/73	1.36%
Constipation	1/73	1.36%
Distension	0/73	0%
Diarrhea	0/73	0%
Vomiting	0/73	0%
32. Has your child had any sickness requiring medication (like antibiotics), such as flu, cold or urinary tract infection, in the past month		
Yes	4	5.5%
No	69	94.5%
33. Which of the following vaccines has your child had in the last year?		
Influenza vaccine	0	0%
Pneumococcal vaccine	0	0%
Neither of these	73	100%
34. Has your child’s sleep pattern and quality changed in the past month?		
Increase in sleep duration	6	8.2%
Decrease in sleep duration	5	6.8%
Change in sleep pattern	32	43.8%
No effect on sleep pattern	30	41.1%
